# Showcasing the role of seawater in bacteria recruitment and microbiome stability in sponges

**DOI:** 10.1038/s41598-018-33545-1

**Published:** 2018-10-12

**Authors:** Marta Turon, Joan Cáliz, Leire Garate, Emilio O. Casamayor, Maria J. Uriz

**Affiliations:** Centre d’Estudis Avançats de Blanes, CEAB-CSIC, Accés Cala St. Francesc, Blanes, Girona, 17300 Spain

## Abstract

We studied the core bacterial communities of 19 sponge species from Nha Trang Bay (Central Vietnam), with particular emphasis on the contribution of planktonic seawater bacteria to the sponge core microbiomes. To ensure consistent sponge-microbe associations and accurate identification of planktonic bacteria transmitted from seawater, we were very restrictive with the definition of the sponge core microbiomes (present in all the replicates), and with the identification of valid biological 16S rRNA gene sequences (100% sequence identity) that belonged to potentially different bacterial taxa. We found a high overlap (>50% relative abundance) between the sponge species core microbiome and the seawater bacterial core in ca. a half of the studied species, including representatives of both, HMA and LMA sponges. From our restrictive analysis, we point to horizontal transmission as a relevant way of symbiont acquisition in sponges. Some species-specific recognition mechanisms may act in sponges to enrich specific seawater bacteria in their tissues. These mechanisms would allow the maintenance of bacterial communities in a species across geographical ranges. Moreover, besides contrasting preferences in bacteria selection from seawater, divergent physiological traits may also account for the different microbiomes in species of HMA and LMA sponges.

## Introduction

The first step to study multi-microbial symbionts within animals is to focus on permanent symbionts by ruling out the background noise produced by transient microbes^1^. In other words, to concentrate on those bacteria, which have established tight associations with the host through several evolutionary time scales^2^, independently of potential, mutual benefits or costs for the partners involved^3^. In this context, the core microbiota concept was adopted to ascertain the consistent associations of a metaorganism^4,5^, but also allows to study the core metabolic functions provided by the host–microbe interaction to the system^6^. The core concept was first applied to differentiate host–microbe interactions of the mammalian gut and in plant root systems^5,6^. Further, it was extended to marine animals with the aim of understanding the consistent contributions of the microbial symbionts to the host ecology, success, or decay^1^.

In marine habitats, studies on coral and sponge microbiomes have proliferated in the last 10 years. For instance, the persistent microbial symbionts (core) of several coral species from a reef were identified^7^ through spatial and temporal scales, concluding that the complexity of the reef habitat, and the life coral history traits likely influence the coral core microbiomes. Thus, more in deep research to explore accurately the core microbiome of many invertebrates is needed to overcome the constraints associated to their complex habitats. Although there is some controversial in the literature about how the core microbiota should be defined^1,8^, the most reasonable definition always depends on the question approached^9^. For instance, more or less restrictive criteria (i.e. present from 7% to 100% of the replicates) have been used for marine invertebrates^1,5,10,11^.

Sponges are a diverse group of sessile filtering invertebrates that play important ecological roles in benthic marine ecosystems^12,13^. They harbour the highest diversity of microsymbionts among marine invertebrates^14,15^. A wide range of studies using massive sequencing methods have retrieved thousands of microbial 16S rRNA gene sequences for each targeted sponge species^11^, which stressed the difficulty to unveil the mechanisms underlying these associations due to the high diversity of the partners involved. Indeed, some of the 16S rRNA gene sequences belonged to transient bacteria captured from the environment by the sponges while filtering seawater, and do not represent permanent symbionts^11^. Consequently, indirect analytical methods have been implemented trying to split stable symbionts from transient planktonic bacteria in sponge-microbial systems. The core microbiota concept was adopted to focus on permanent bacteria in a sponge species^11^ or phylum^10^, notwithstanding the host geographical and ecological origins, or temporal scales^16^. The rationale underlying the core concept is that stable symbionts should represent tight biological associations and thus, they are expected to be present in most, or all, host individuals.

Substantial differences in the resulting diversity metrics related to the core definition applied (from 12% to 100% occurrence), have been reported recently^9^. Several studies have proposed that stable sponge microsymbionts should be present in the sponges but not (at least not in high abundance) in the surrounding seawater^11^. But, recording the presence of a sponge bacterium in seawater greatly depends on the abundance threshold used to include or exclude sequences from downstream analysis, the sequencing depth and the number of samples. Moreover, it has been proposed that stable symbionts are mainly inherited by the progeny from their parent sponges^17–20^. However, vertical and horizontal transmission of the same bacteria have been described^21^ and more recent investigations reported different microbiomes in adults and larvae of the same species, which highlights the role of seawater bacteria in the structural composition of sponge microbiomes^17,22^.

Differences in microbial diversity between High Microbial Abundance (HMA) and Low Microbial Abundance (LMA) sponges have been reported^23,24^. These differences have been related to contrasting structural and physiological traits of the two species groups^25^. HMA sponges have a denser mesohyle and a more complex aquiferous system, with smaller choanocyte chambers, than LMA sponges^25^. Also a partial trophic niche separation for HMA and LMA sponges has been proposed^26^. Comparisons of the core microbiomes of these two groups, which consisted in bacterial T-RFLPs that were present in all species replicates across seasons and study years, showed that HMA sponges had a larger number of core bacterial groups with a higher overlap with seawater than LMA hosts^27^.

In this study, we attempted to cast some light on the acquisition modes of microbial symbionts in sponges by i) unveiling the permanent microbiomes within a large number of sponge species, and ii) estimating the contribution of seawater bacteria to the sponge core microbiomes in representatives of both HMA and LMA sponges. To address these goals, we analysed the sponge microbiomes of the 19 most abundant sponge species inhabiting a small geographical area in Nha Trang Bay (Central Vietnam), as well as the bacterial assemblages of the surrounding seawater. We applied a restrictive approach to core community concept in terms of bacteria occurrence across species replicates and to OTU definition. Sequence identity thresholds <99% for the 16S rRNA V4 region have been proved to be inaccurate for bacterial species delimitation^28^, in particular for short reads obtained from Next Generation Sequencing (NGS). Instead, a 100% of sequence identity has been proposed to obtain true biologically informative sequences, which can underlie metabolic and ecological particularities^28^. To ensure that the 16S rRNA gene sequences recovered from the seawater samples were identical to those recovered from the sponges, we clustered OTUs (Operational Taxonomic Units) at 100% identity (Zero radius OTUS or ZOTUs^28^), which has only been recently applied in a couple of studies of sponge microbiomes^29,30^. Clustering sequences at 100% identity and restricting the core to microbes present in 100% of the analysed samples seem particularly relevant when trying to elucidate horizontal symbiont acquisition.

## Results

### Specificity of sponge bacterial communities

The main factor structuring the sponge microbiomes was the sponge species (Permanova: R^2^ 0.56, p-value < 0.01), which means that replicates from the same species were more closely related to each other than to any other species. However, dispersion within replicates greatly varied depending on the sponge species (Permutest F: 6.9 p-value < 0.01). A dichotomy (Permanova: R^2^ 0.11, p-value < 0.01) between the so-called HMA and LMA sponges regarding their bacterial composition was detected (Supplementary Fig. S1). Moreover, dispersion within the three HMA species (*Aaptos suberitioides, Neofibularia hartmani* and *Suberea cf. laboutei*) was much lower than the dispersion within the 16 LMA species (Permutest F: 60.8 p-value < 0.01, Supplementary Fig. S1).

### Core communities and species specific ZOTUs

The ZOTU richness of the core communities (ZOTUs present in all replicates of the same sponge species) varied from 54 in *Clathria reinwardti* to 600 in *Thrinacophora cf. raphidophora* (Table 1). The number of species replicates influences the number of ZOTUs forming the core community, the more replicates taken into account, the lowest the number of core ZOTUs (R_S_ = −0.83, p-value < 0.01, Supplementary Fig. S2A). However, ZOTU abundance of the sponge species core did not depend on the number of species replicates since no correlation was found between both variables (R_S_ = −0.21, p-value > 0.05, Supplementary Fig. S2B

**Table 1:** Mean, core, species-specific, and SW ZOTUs of all species studied. Values are given in number and relative abundance (%) of ZOTUs. HMA species are marked in dark grey and LMA species are marked in light grey. (n = number of replicates per species).

Species	n	Mean ZOTUs ±SD	Core ZOTUs	% Ab. Core ±SD	Sp-sp ZOTUs^(a)^	% Ab. Sp-sp ZOTUs	SW ZOTUs^(b)^	% Ab. SW ZOTUs
*Aaptos suberitoides (Brondsted, 1934)	13	740 ± 139	134	77.6 ± 6.2	6	1.1	53	48.79
*Neofibularia hartmani (Hooper & Lévi, 1993)	10	883 ± 182	167	94.2 ± 1.6	32	4.4	67	71.59
*Suberea cf.laboutei (Bergquist, 1995)	3	812 ± 133	206	79.2 ± 7.8	65	20.6	61	26.67
*Amphimedon paraviridis* (Fromont, 1993)	10	587 ± 223	57	61.9 ± 22.1	1	0.0	52	99.54
*Antho* sp.	3	1587 ± 872	404	88.2 ± 5.1	71	2.0	107	9.90
*Callyspongia* sp.	2	850 ± 507	229	83.9 ± 2	30	1.0	76	38.55
*Clathria reinwardti* (Vosmaer, 1880)	15	769.1 ± 171	54	88.4 ± 7.3	0	0.0	36	90.40
*Clathria* sp.	4	838 ± 420	144	69.6 ± 19.9	2	0.3	50	33.57
*Dendroxea* sp.	2	795 ± 118	310	77.7 ± 13.9	50	11.6	99	33.99
*Dysidea* sp.	3	1811 ± 902	453	84.6 ± 6.8	66	3.7	131	27.35
*Gellioides cf.gracilis* (Hentchel, 1912)	9	647 ± 123	138	82.9 ± 5.3	1	0.4	69	53.03
*Gellioides* sp.	4	456 ± 87	96	49.3 ± 27.5	8	25.7	83	93.22
*Haliclona* sp.	3	1343 ± 611	365	79.5 ± 9.7	44	3.1	123	16.06
*H. (Gellius) toxotes* (Hentchel, 1912)	3	892 ± 327	249	74.3 ± 4.2	15	0.2	88	60.34
*Monanchora unguiculata* (Dendy, 1922)	3	622 ± 115	254	86 ± 7.8	12	0.3	93	58.71
*Mycale* sp.	4	1720 ± 1576	80	54 ± 7.3	2	0.7	42	77.51
*Phorbas* sp.	3	905 ± 349	214	86.6 ± 6.6	14	0.6	77	52.20
*Pseudosuberites* sp.	2	1791 ± 1205	579	86.4 ± 9.4	171	4.3	125	10.97
*Thrinacophora rhaphidophora* (Hentschel, 1912)	2	1392 ± 231	600	89.1 ± 5.2	195	9.6	169	22.67

*HMA species.

^(a)^Number and percentages of abundances of species-specific ZOTUs are calculated for the core community of each species.

^(b)^Number and percentages of abundances of SW ZOTUs are calculated for the core community of each species. Values correspond to the comparison with the SW core ZOTUs (cosmpolitan).

). This means that the abundant ZOTUs are the major contributors to the core of a species, and that the variable fraction is represented by the low abundance ZOTUs. Thus, we have considered the comparisons based on the relative abundances of the core ZOTUs.

**Figure 1:**
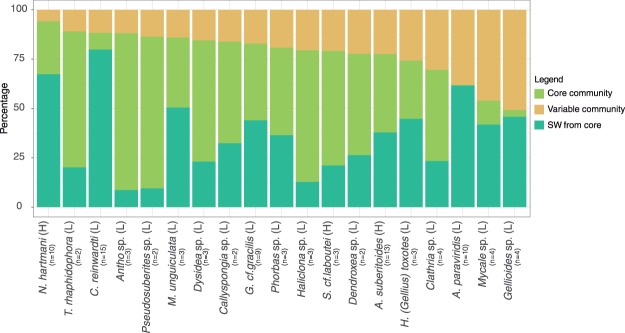
Mean relative abundances for the core (green) and the variable fraction (yellow) of the sponge microbiomes. Blue lines delimit the percentage of the sponge bacterial core shared with the seawater bacterial community. H = High Microbial Abundance sponge, L = Low Microbial Abundance sponge.

The core community represented more than 75% of the reads from the total microbiome in most species (Fig. 1). *N. hartmani* was the species with the largest core microbiome, representing up to 94% of relative abundance (Table 1) and *Gellioides* sp. had the smaller core microbiome representing <50% of relative abundance.

The number of species-specific ZOTUs (those present in all replicates of a species and absent from the core of any other study sponge species) varied from 0 (in *C. reinwardti*) to 195 (in *T. cf. raphidodophora*). However, the abundance of species–specific ZOTUs did not surpass 25% in any case, and most often were below 5% of relative abundance in the respective core communities. *Gellioides* sp. and *S. cf. laboutei* were the species with the highest relative abundances of species-specific ZOTUs (25% and 20%, respectively).

Similar values of core communities and species-specific bacteria were obtained for the dataset analysed with OTUs clustered at 97% sequence similarity (Supplementary Table S1).

### Composition of the bacterial core communities

**Figure 2:**
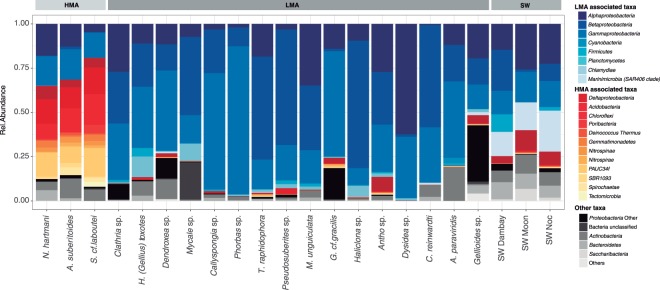
Mean relative abundance of core microbial taxa at a phylum level (class for *Proteobacteria*) within each sponge species and seawater samples. Bacterial taxa with significant Indval values (Supplementary Fig. S3) associated to HMA species are marked in reddish colours and the ones associated to LMA species are marked in bluish colours.

Specific associations between certain bacteria (phylum and class level) and HMA or LMA species were detected with the *Indval* analysis (Supplementary Fig. S3). These indicator bacteria are present in the core communities of the studied sponges (Fig. 2). The three HMA species presented an alike core microbial composition and abundances at phylum level with specific bacterial phyla overrepresented, compared to LMA species and seawater (SW). Phyla associated with LMA sponges also showed a similar core microbial composition but with contrasting abundances in the several species. Seawater samples had their own core bacterial composition with representatives of bacterial phyla shared with either HMA or LMA sponges. Mean Shannon diversity indices of the species core communities were significantly higher (Kruskal-Wallis <0.01) for HMA species (3.8 ± 0.24) than for LMA species (2.3 ± 0.61) (Supplementary Fig. S4).

### Seawater (SW) ZOTUs

**Figure 3:**
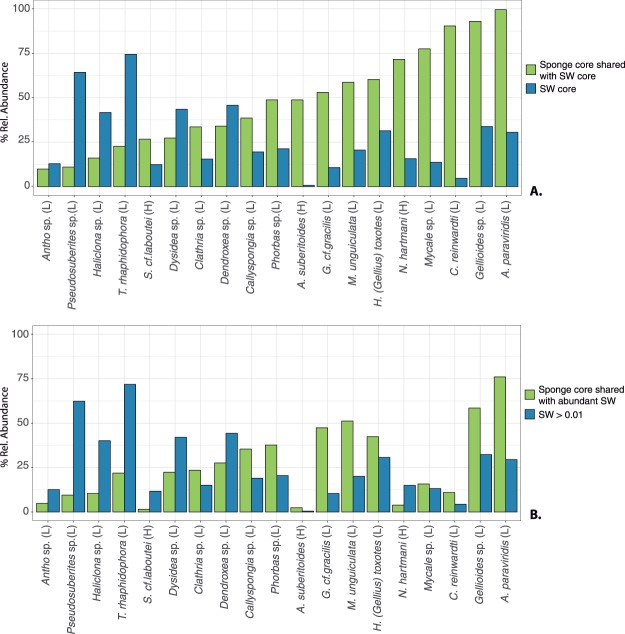
Mean relative abundances of shared bacteria between the SW and the core microbiomes for each sponge species when comparisons were made with the (**A**) SW core (cosmopolitan bacteria) and (**B**) the abundant (>0.01%) SW bacteria. Bars represent percentages of relative abundances of the shared bacteria in both, the sponge (green colour) and the SW (blue colour). H = High Microbial Abundance sponge, L = Low Microbial Abundance sponge.

The relative abundances of the shared core SW ZOTUs (detailed in the Experimental procedures section) with the sponge core microbiomes varied between 9% and 99% depending on the sponge species (Fig. 3A). The sponges with the highest contribution of core SW ZOTUs to their core microbiome were *A*. *paraviridis* (99.5%), *Gellioides* sp. (93.2%) and *C. reinwardti* (90.4%), while *Antho* sp. (9.9%) and *Pseudosuberites* sp. (10.9%) showed the lowest overlap between the sponge and the SW core bacteria (Fig. 3A, Table 1). In some species (i.e.: *A. suberitoides, C. reinwardti*, *Mycale* sp.), rare SW ZOTUs (>0.01% of relative abundance) were abundant in the core of the sponge species. This is particularly visible in the example of *C. reinwardti*, which harboured ZOTUs that represented just a 5% of the SW core community and 90% of the sponge core. The opposite occurred in other sponge species such as *Pseudosuberites* sp. and *T. cf. raphidophora*, which had highly abundant (>1% of relative abundance) SW ZOTUs poorly represented in their core.

**Figure 4:**
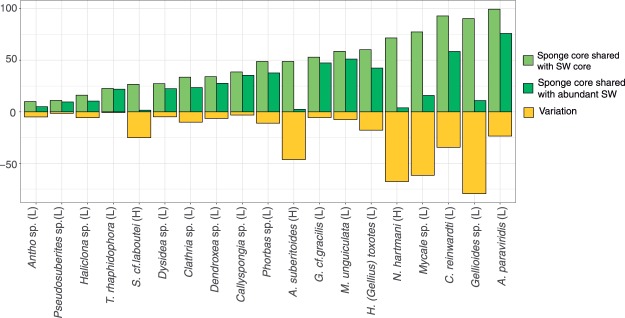
Variation in relative abundance of SW ZOTUs in the sponge cores according to the method used for comparisons. Light green bars show ZOTUs shared with the SW core. Dark green bars represent ZOTUs shared with the abundant SW ZOTUs. Negative bars (yellow) represent the differences in shared ZOTUS between methodologies. H = High Microbial Abundance sponge, L = Low Microbial Abundance sponge.

In addition, when the most abundant SW ZOTUs were considered (abundances higher than 0.01%), instead of the SW core ZOTUs, the relative abundances of bacteria shared with SW were drastically reduced in most of sponge core microbiomes, with most values below 40% (Fig. 3B). This reduction was notably relevant in some species, such as *Gellioides* sp. *N. hartmani, Mycale* sp*. A. suberitioides, C. reinwardti*, and *S. cf. labutei* (Fig. 4).

Results of both comparisons for OTUs at 97% sequence identity are shown in Table S2. Differences between OTUs and ZOTUs were more remarkable when considering the abundant SW ZOTUs. For instance, in the case of *C. reinwardti*, the proportion of SW OTUs changed from 10% (for ZOTUs) to 73% (for OTUs). Also remarkable were the differences for *A*. *paraviridis*, *Gellioides* sp., and *Phorbas* sp.

**Figure 5:**
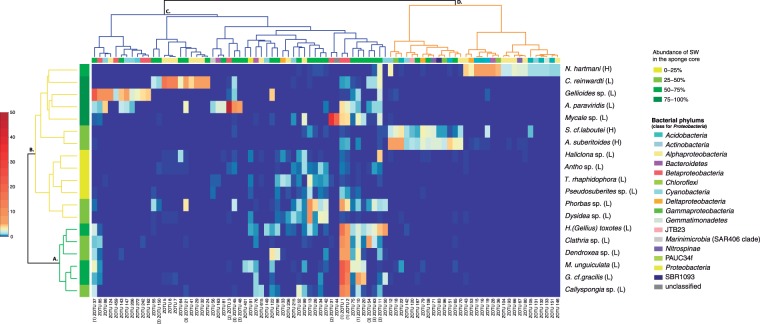
Heatmap showing core (cosmopolitan) SW ZOTUs with relative abundances higher than 1% across sponge species (listed on the right side). On the left, hierarchical clustering using Bray-Curtis dissimilarity matrix of the sponge species (colours on the vertical stripe represent the relative abundance of SW ZOTUs). On top, hierarchical clustering using Bray-Curtis dissimilarity matrix of the bacterial ZOTUs (colours on the horizontal stripe indicate ZOTU taxonomy at a Phylum level (class level for *Proteobacteria*). ZOTUs abundance is represented in the colour temperature bar on the left. Letters A, B, C, D indicate the different clusters. ZOTU numbers between parenthesis means: (1) ZOTUs shared by cluster A, (2) *Candidatus Branchiomonas* ZOTUs, (3) *Endozoicomonas* ZOTUs. H = High Microbial Abundance sponge, L = Low Microbial Abundance sponge.

The abundance and distribution of the SW core ZOTUs in the sponge core microbiomes is shown as a heatmap representation (Fig. 5). Two sponge clusters (A and B) were differentiated in the dendrogram. Cluster A contained sponge species that shared two highly abundant SW ZOTUs (ZOTU1, ZOTU2 at mean abundances of ~10%), and some ZOTUs (10, 37) at abundances higher than 1%. Cluster B comprised species harbouring different SW bacteria at contrasting abundances. Two different ZOTUs of *Candidatus Branchiomonas* (*Betaproteobacteria*) accounted for more than 30% and 50% of the *Mycale* sp. and *A*. *paraviridis* core microbiomes, respectively. Similarly, ZOTUs belonging to several *Endozoicomonas* (*Gammaproteobacteria*) were present at different relative abundances in the four species with the highest proportion of SW ZOTUs (*Gellioides* sp, *A*. *paraviridis*, *Mycale* sp. and *C*. *reinwardti*). Two main groups (C, D) were also differentiated according to the bacterial classes. The first group (C) corresponded to bacterial classes associated to LMA sponges, with high abundances of *Alpha-, Beta-*, and *Gamma- Proteobacteria*. The second group (D) showed bacterial classes associated to HMA sponges. ZOTUs belonging to PAUC34f, *Chloroflexi, Acidobacteria, Actinobacteria*, and *Nitrospinae* were almost exclusively found in the three HMA species (*N*. *hartmani*, *S. cf. laboutei* and *A. suberitioides*).

## Discussion

The sponge-associated bacteria were species specific for the 19 study species, as previously reported for sponges from other geographical areas^10,17,25,31–34^. Selection of specific bacteria and competition among the selected bacteria^31^ may converge to shape the species-specific patterns of sponge microbiomes, which used to be similar in geographically distant individuals of the same genus^35^.

However, differentiated patterns of bacterial composition are observed according to the affiliation to the HMA or LMA groups, as previously reported^36,37^. Indicator bacteria at class level for both groups are found, which match the indicator classes inferred from differential abundance analysis^38^. It has been reported that HMA sponges show higher microbiome similarity among species than LMA sponges^27^. Moreover, bacterial diversity is also claimed to be higher in HMA^14,23,27,39,40^. These aspects are confirmed in our study when considering the core members of both HMA and LMA sponges, although the number of representatives of each group is unbalanced.

It has been assumed^40,41^, but also questioned^24,25^, that LMA sponges contain mainly transient seawater bacteria. Representatives of both HMA and LMA study sponges contained high percentages of SW core bacteria (>50% of core relative abundance). However, species of each group acquire different SW bacteria that are indicators of either HMA or LMA sponges (Fig. 2 and Supplementary Fig. S3), suggesting contrasting bacteria selection mechanisms in each group. Differences might also be enhanced by particular traits of the respective physiology of the two sponge types^42^.

To define species core and species-specific bacteria, we clustered sequences at 100% identity (ZOTUs). Only recently, the sponge microbiomes have been analysed at the ZOTU level^29,30^. By recording ZOTUs, we were able to identify closely related bacteria^28^, which may inhabit sponge and seawater biomes making comparisons more reliable.

Analysing the bacterial core at species level provides information about stable, purportedly fixed associations, which may be involved in sponge-bacteria interaction patterns. To define the species core, an appropriate percentage of bacteria occurrence across species replicates has to be selected depending on the study aims^9^. For example, 85% occurrence was used for a species with more than 47 replicates to conduct an interaction network analysis^11^. In our study, as we wanted to focus on the truly symbiotic bacterial community of each sponge species, we choose a restrictive approach to the species core by only considering bacteria that were present in all the replicates of each species (100% occurrence), disregarding whether they were present among the SW core or not. In this way we ensured that we were focusing on persistent symbionts rather than on transient microbes.

The size of the bacterial core, which represents the permanent part of the sponge microbiome, seems to be intrinsic of each sponge species^16^. A high stability of the microbiome across sponge replicates can be indicative of the strength of the sponge-bacteria associations, whereas the opposite would indicate the presence of facultative /transient bacteria^11^. Most of the associations among bacteria and the studied species appeared to be highly constant, thus, suggesting a strongly fixed relationship, although we are aware that this might depend on the number of replicates analysed.

The contribution of the species-specific ZOTUs to the core communities, in terms of relative abundance, was surprisingly low. Those values may be influenced by the number of sponge species taken into account in the study, the similarity between their microbiomes, and, more strongly, by the restrictions associated to the way species-specific OTUs are defined, whether being part of the species core community (our study) or not^10,32^. Schmitt *et al*.^10^ suggested that the species-specific bacteria would probably be vertically inherited. If this assumption is true, the percentage of vertically inherited symbionts in our sponges would be rather low, since we have found a few species-specific microbes and the majority of the sponge bacterial taxa are found in more than one species.

Both, sponge microbiomes and seawater communities are in close contact because of the filtering activity of sponges, which can result in occasional bacteria transfer from one source to another^24^. To avoid this potential contamination, we used the 100% occurrence core approach in both the sponges and seawater, because it is unlikely that a ZOTU contaminates all the replicates of the same source. Moreover, we took into account the differential abundances that a ZOTU can be present in both, the sponge and the seawater. In this way, to be conservative, we could only suspect of SW bacteria contamination in the cases in which abundant SW bacteria are in low abundance in a sponge for which only few replicates are available. These cases would merit further investigation.

Our results comparing the bacterial core of sponges and seawater showed that all the studied sponges contained SW bacteria, as previously reported for other sponge species^24,32^. However, the relative abundance of SW bacteria in the sponge microbiomes was species dependent, ranging from almost 100% in *A. paraviridis* to less than 10% in *Antho* sp.

The quantification of the relative abundance of SW bacteria in the sponge microbiomes is particularly relevant to address seasonal or geographical changes and species specific traits^24^, but also allows inference about bacterial transmission modes. Thus, we estimated the relative abundance of the SW- sponge shared bacteria in each biome core trying to differentiate SW microbes that may represent stable symbionts from transient contaminant bacteria. We postulated that enrichment of seawater bacteria in the sponge occurred when low abundance SW core bacteria were found as the main components of the sponge microbiomes (Fig. 3). This can be proposed for 11 out of 19 sponge species analysed and in particular, for *C. reinwardti*, *A. paraviridis, Mycale* sp*., Gellioides* sp*. N. hartmani*, and *A. suberitoides*. No pattern related to the HMA and LMA dichotomy could be withdrawn here, as representatives of both groups showed enrichment of seawater bacteria in their microbiomes. The proportion of the sponge core bacteria shared with SW is reduced drastically in many species when comparisons are performed with the abundant SW bacteria, instead of with the SW core bacteria. Among these species, the three HMA species (*N. hartmani, A. suberitioides*, and *S. cf. laboutei)*, reduced their relative abundance of SW ZOTUs from ~50%, ~70% and ~25%, respectively, to values below 5% (see Fig. 4). Reduction occurs in sponges that harbour low-abundance SW bacteria that would be ignored when only abundant SW bacteria are used for comparisons. Therefore, the study HMA sponges contain in their microbiomes SW bacteria that are at low-abundance in the water. For species that harbour both abundant and SW core bacteria in their microbiomes, similar percentages of SW bacteria were obtained with both methodologies. With this comparison, we like to point out how different approaches may influence the results on the overlap between the sponge and SW microbiomes. In particular, we emphasize the importance of the approach used for studies aiming to elucidate the dichotomy between HMA and LMA sponges.

We considered relevant the contribution of SW bacteria to the formation of the sponge microbiome when they were present across all species replicates. In contrast, some authors^11^ considered that abundant (>0.01%) SW OTUs likely represent environmental contaminants and should be removed from the sponge samples, independently of their abundance in the sponge. Conversely, we propose, that only highly abundant SW bacteria that are rare in the sponge-species core are potential candidates to represent SW bacteria contamination, especially when only few replicates are available. Two sponge species from our dataset (e.g. *Pseudosuberites* sp. and *T. raphidophora*) are examples of possible contamination.

Overall, we consider the comparisons using the SW core bacteria as a more accurate way to assess the true sponge-SW shared bacteria, as it considers bacteria that would be available across locations to be incorporated in the sponge microbiome. Our results support that “sponge-specific” bacteria are rather “sponge-enriched” bacterial clusters, and that seawater acts as a seed bank for sponge microbiomes, as suggested by Webster *et al*.^17^, Webster and Thomas^43^ and Moitinho-Silva *et al*.^24^. We detected widespread but rare^44^ SW bacteria forming part of sponge core microbiomes. Whether these taxa are metabolically active in the water column or represent dormant stages that reactivate after being incorporate to the sponge^24^ remains to be elucidated.

We find a high specificity of the associations between sponges and seawater bacteria. Each sponge species seems to incorporate different bacteria from the seawater in its microbiome. This suggests that some species-specific mechanisms have been fixed in the sponges to select some seawater bacteria and not others. Recognition mechanisms have been proposed to explain horizontal acquisition of microbes from the surrounding environment^22^. Taking into account the high percentages of seawater bacteria detected in some of our sponge species and the species-specificity of many of them, we propose that environmental acquisition would play a major role in the establishment of species-specific sponge microbiomes.

To summarize, sponge species is the main factor structuring microbiomes of the most common sponges from Nha Trang bay (Vietnam). By using a very restrictive approach of the “core species” concept and ZOTUs with 100% sequence identity for defining bacterial species, we proved that intra-species microbiome stability is the rule for most sponges. A high percentage of SW bacteria shaped the core microbiome in many study species. Our results point to horizontal transmission, as an ubiquitous mechanism of symbiont acquisition in sponges, while vertical transmission would represent a rather complementary acquisition way. Apparently, some highly specific recognition mechanisms may be acting in sponges to specifically enrich some SW bacteria in their tissues, and not others. Moreover, contrasting preferences in bacteria selection may account for differences in the microbiomes of HMA and LMA sponges and some physiological traits such as contrasting filtration rates might also contribute to enhance the differences. These mechanisms would allow the maintenance of stable bacterial communities disregarding environment conditions and geographical distance and merits to be confirmed by analysing in the same way as in the current study a larger number of sponge and water samples from different geographical regions.

## Experimental Procedures

### Sponge and seawater sampling and DNA extraction

Sponge samples were collected in April 2015 by SCUBA diving along 13 transects, 25 m long each, randomly placed between 3 and 9 m deep in three neighbouring locations ~2 km apart (i.e.: Dambay, Hun Mun and Nock Island) within Nha Trang Bay (central Vietnam). This quantitative sampling method allowed us to detect the most representative sponges in the study area but not to collect the same number of replicates for all the species. For instance, only three HMA sponges were found in the whole sampling but two of them were present at high abundances. Overall, we collected 203 sponge samples, from which we only considered for this study the ones that were found at least twice. Thereby, 98 samples belonging to 19 sponge species with between 2 and 15 replicates each (Table 1) were analysed.

Each sponge individual fitting within a transect was photographed and a piece of ca. 3 cm^2^ was collected in a 50 mL Falcon tube in seawater. Seawater was immediately replaced by 100% ethanol once on board. Back in the lab, the ethanol was replaced twice again with fresh absolute ethanol for a good sample preservation. DNA from those sponges was extracted following the protocol of DNeasy Blood &Tissue Kit (Qiagen).

Triplicate plankton samples were taken from the three sampling locations where the ecological transects were performed (i.e.; Dambay, Hun Mun and Nock Island). Two litres of water were collected and sequentially filtered throughout 5 μm, to remove undesired plankton components, and then throughout 0.22 μm polycarbonate membranes. The size fraction between 5 and 0.22 μm was processed for DNA extraction. Membranes were enzymatically digested with lysozyme, proteinase K and sodium dodecylsulfate and afterwards, DNA was extracted with phenol:chloroform-isoamyl alcohol (25:24:1, vol/vol/vol) and chloroform:isoamyl alchohol (24:1, vol/vol). Purification and concentration of the DNA was carried out with Amicon® Ultra 4 Centrifugal Filter Units – 100000 NMWL (Millipore). The extraction procedures used for sponge samples and SW were the most appropriated according to their respective preservation.

### Sponge identification

We identified sponge species to the best possible taxonomic resolution by molecular markers and morphological features. Fragments of the nuclear genes encoding the 18S rRNA (~1700 bp) and 28S rRNA (~650 bp), as well as the cytochrome c oxidase subunit I (*COI* ~680 bp) were amplified and sequenced. Primers 1 F and 1795R^45^ were employed to amplify 18S rRNA, Por28S-830F and Por28S-1520R^46^ primers were used to amplify the D3-D5 partition of the 28S rRNA and LCO1490 and HCO2198^47^ were used for *COI*. PCR amplifications were conducted in 50 μl reactions containing 1 ng of template genomic DNA, 5 μl of 10x PCR buffer (containing 1.5 mM MgCl_2_), 2 μl of dNTP mix (10 mM), 2 μl of bovine serum albumin, 1 μl pf each primer (10 mM) and 0.4 μl of Taq DNA polymerase (5 U μl^−1^). The temperature profile for the 18S rRNA was as follows: 94 °C/5 min; (94 °C/1 min, 50 °C/1 min, 72 °C/1 min) × 35 cycles; 72 °C/5 min; for 28S rRNA: 94 °C/5 min; (94 °C/30 s, 53 °C/30 s, 72 °C/30 s) × 30 cycles; 72 °C/5 min; and for COI: 94 °C/2 min; (94 °C/1 min, 45 °C/1 min, 72 °C/1 min) × 35 cycles; 72 °C/7 min. Purification and sequencing were carried out by an external service (Macrogen, Netherlands). The obtained sequences were manually edited in Geneious v 9.0.2 and blasted against NCBI databaste (https://blast.ncbi.nlm.nih.gov/Blast.cgi) to confirm the morphological identification of the sponges at the lowest taxonomic level possible.

Preparation of spicules and histological sections were made from specimens’ subsamples and observed under both, light and scanning electron microscopes. Morphological characters such as spicule types, shape, length, and width, as well as skeletal arrangement^48^ were used in combination with individual sequences and phylogenetic reconstructions to obtain, the most accurately possible, taxonomic identifications. Sponges were classified as HMA or LMA on the basis of their pertinence to genera already known to belong to any of these two groups, what was additionally confirmed by looking to the characteristics and structure of the sponge aquiferous system: small, relatively few (HMA) *vs*. large abundant choanocyte chambers (LMA), and mesohyle (dense *vs*. lax mesohyle, respectively)^25^.

### 16S rRNA gene amplification, sequencing and analysing

PCR and high-speed multiplexed SSU rRNA gene Illumina MiSeq sequencing (NGS) were carried out following the genomic core facilities and methods of the MrDNA Lab (Texas, USA) (http://www.mrdnalab.com/). The variable V4 region of the 16S rRNA gene (c.a. 250 nt) was amplified using the primers 564F (5′AYTGGGYDTAAAGNG3′) and 785R (5′TACNVGGGTATCTAATCC3)^49^. Raw rRNA gene sequences were processed using the UPARSE pipeline^50^. A quality check and de-replication were applied to our dataset. Denoising (error-correction) of amplicons was performed to identify all correct biological sequences following the UNOISE pipeline^51^. This algorithm removed chimeras, reads with sequencing errors, PhiX, and low complexity sequences due to Illumina artifacts, and generates ZOTUs (“zero-radius” OTUs) consisting of sequences of 100% identity. For comparison purposes, sequences were also clustered at 97% threshold (Supplementary information). For this analysis, reads were dereplicated and clustered into operational taxonomic units (OTUs) at cut-off 0.03% identity after chimera removal (UCHIME) and excluding the singletons.

Taxonomic assignment was done with SINA v1.2.11^52^ using SILVA 128 database. SINA uses Lowest Common Ancestor method (LCA). We configurated a “Min identity” of 0.7 and a maximum number of search results of 1 per sequence results in “best match” type. Sequences with low alignment quality (<75%) and sequences identified as mitochondria or chloroplasts were removed from the analysis. In order to minimize biased effects for differences in sampling effort, the original ZOTU table was rarefied (Supplementary Fig. S5) at a minimum reads threshold of 41000^53^.

Raw sequences are available in the SRA archive under the project number PRJNA453898.

### Defining core and species-specific ZOTUs

We identified the ZOTUs that were present in all replicates to define the core microbiome of each sponge species. The ZOTUs that did not meet this requirement were assigned to the variable community. Moreover, we considered species-specific ZOTUs those belonging to a single core microbiome for a particular sponge species, compared to the remaining collected sponges.

### Seawater (SW) ZOTUs

We combined two approaches to estimate the real contribution of the seawater (SW) bacteria to the sponge core microbiomes. First, we looked for bacteria in each sponge species that were already present in the core SW community. We considered the core community of the SW as the community formed by the ZOTUs present in all water replicates. With this approach, we attempted to identify bacteria that commonly inhabited in the SW and that its presence was not merely circumstantial. Therefore, they could represent a potential source for the formation of the sponge microbiome over time. In the second approach, we made the comparison with the most abundant bacteria of the SW. We identified the ZOTUs with relative abundances higher than 0.01% in average across all water samples. This threshold was chosen since it has been used previously to remove from the sponge microbiome samples the OTUs that were likely to represent environmental contaminants^11^. With this approach, we aimed to gain insight on the SW bacteria that are more likely detected in the sponge microbiomes just because of their high abundance in SW and may represent transient (environmental) contaminants^11^. On the other hand, we considered rare ZOTUs those with a relative abundance <0.01% and highly abundant ZOTUs those with relative abundance >1%^32^.

### Statistical analyses

We carried out a distance-based multivariate analysis at ZOTU level of the microbial communities of the sponges and seawater samples using the *vegan* package^54^ in R. A cluster dendrogram was built using the Bray-Curtis dissimilarity distance matrix of samples to visualize patterns of bacterial community structure in sponges and seawater. We tested the effect of host identity (species) as well as the effect of the HMA/LMA identity, on the structure of microbial communities with non-parametric Permutational Analysis of Variance (PERMANOVA). PERMUTEST was applied to detect differences in the dispersion between groups. A bias correction^55^ for the unequal sample size of HMA and LMA groups was applied in the *betadisper* function of the *vegan* package^54^. P-values of PERMANOVA and PERMUTEST were calculated using 999 permutations and significance cut-off for p-values was 0.05.

The mean relative abundance of the bacterial phyla and classes was calculated for both, HMA and LMA groups. We applied an IndVal analysis using the *labdsv* package^56^ in R to detect potential associations of certain bacterial phyla and classes to any of these two groups. We fixed an IndVal threshold of 0.6 (p-value < 0.01) to consider a bacterial taxon strongly associated to (or Indicator of) HMA or LMA sponges.

## Electronic supplementary material

Supplementary information

## Data Availability

The raw prokaryotic sequences analysed during the current study are available in the SRA archive under the project number PRJNA453898 (https://www.ncbi.nlm.nih.gov/sra/?term = PRJNA453898). SSU and LSU Sponge sequences are available under the accession numbers MH731279 to MH731308. COI sponge sequences are available under the accession numbers MH784603 to MH784613. Moreover, the results of the dataset analysed with OTUs clustered at 97% sequence similarity are available in the Supplementary material of the current paper.
