# Supplementary material for: Showcasing the role of seawater in bacteria recruitment and microbiome stability in sponges
Source: Sci Rep. 2018 Oct 12;8:15201. doi: 10.1038/s41598-018-33545-1 (PMC6185911; doi:10.1038/s41598-018-33545-1)
Supplement: Supplementary file 1 — Supplementary information [file 41598_2018_33545_MOESM1_ESM.pdf]

Showcasing the role of seawater in bacteria recruitment and microbiome stability in sponges

Marta Turon, Joan Cáliz, Leire Garate, Emilio O. Casamayor, and Maria J.Uriz

Centre d'Estudis Avançats de Blanes, CEAB-CSIC, Accés Cala St. Francesc, Blanes, Girona,

17300, Spain. mturon@ceab.csic.es; Iosune@ceab.csic.es

Supplementary information

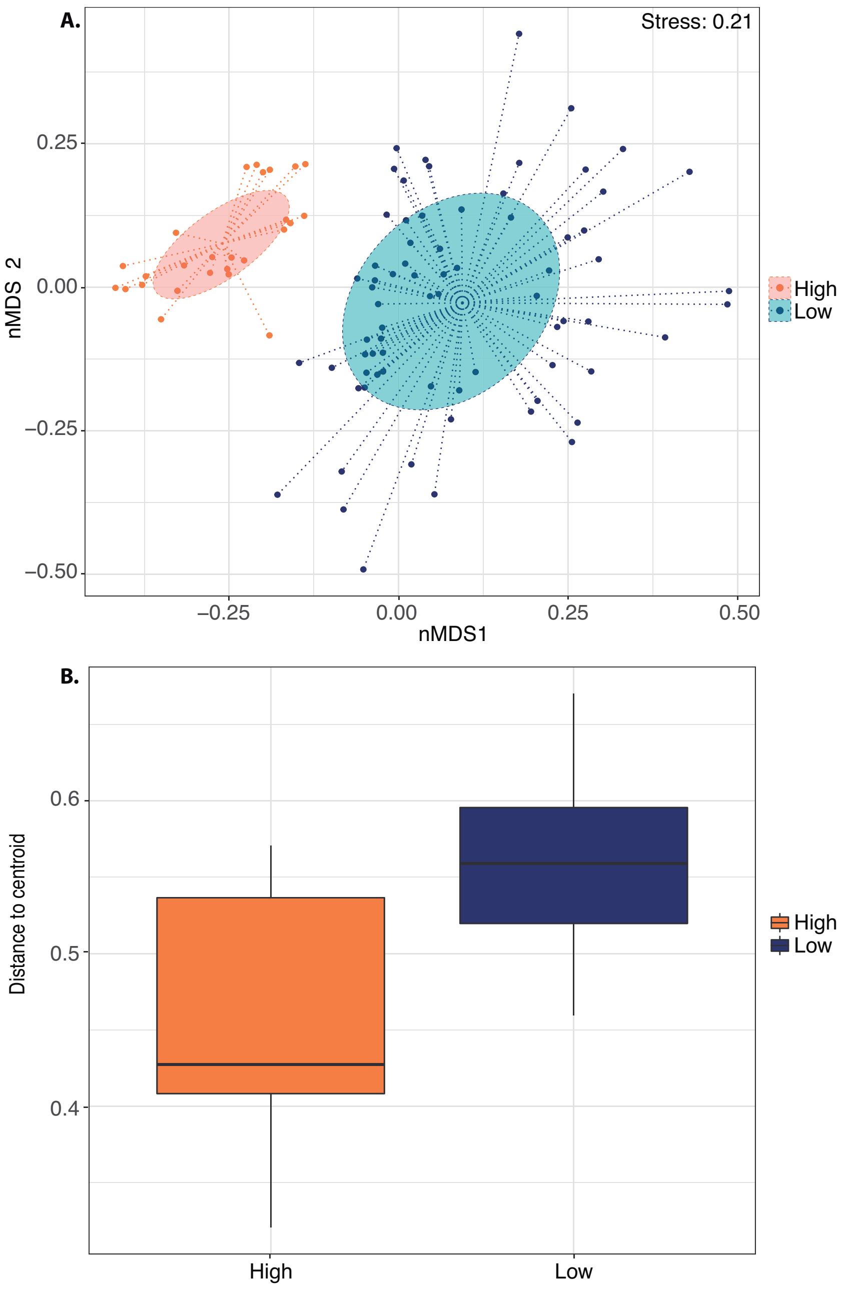

Figure S1. (A) Non-metric multidimensional scaling (nMDS) ordination of the sponge bacterial communities based on Bray-Curtis distances coloured by their belongingness to HMA (orange) or LMA (blue) species. (B) Comparison of the beta dispersion between groups of HMA and LMA species, values represent the distance to centroid of the replicates for each group.

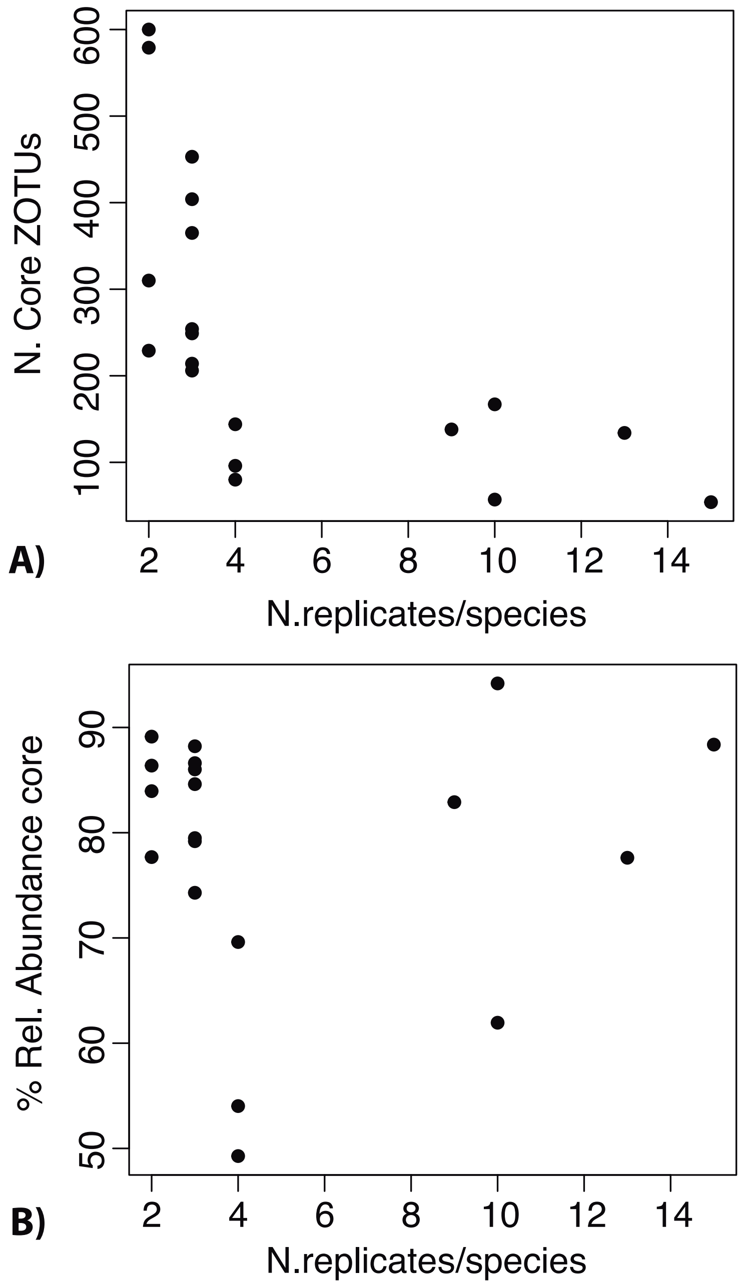

Figure S2. (A) Relation between the number of species replicates (from 2 to 15) and the number of ZOTUs that constitute the core community of an species. (B) Relation between the number of species replicates (from 2 to 15) and the percentage of relative abundance that the core ZOTUs represent to the overall microbiome of each species.

Table S1: Mean, core, species-specific, and SW OTUs of all studied species. Values are given in number and relative abundance (%) of OTUs (97% sequence similarity). HMA species are marked in dark grey and LMA species are marked in light grey. (n= number of replicates per species)

Species	n	Mean OTUs ± SD	Core OTUs	% Ab. Core ± SD	Sp-sp OTUs (a)	% Ab. Sp-sp OTUs	SW OTUs (b)	% Ab. SW OTUs
Aaptos suberitoides (Brondsted, 1934)	13	776 ±100	119	81.2 ± 4	4	0.5	47	56.6
Neofibularia hartmani (Hooper&Lévi, 1993)	10	969 ±101	164	93.2 ± 1.4	45	4.7	68	75.4
Suberea cf.laboutei (Bergquist, 1995)	3	709 ± 50	213	91.7 ± 3.1	64	14.1	67	33.5
Amphimedon paraviridis (Fromont, 1993)	10	524 ±192	48	58.6 ± 20.8	0	0	47	100.0
Antho sp.	3	1214±562	369	89.8 ± 4.3	63	2.1	109	11.2
Callyspongia sp.	2	663 ± 379	203	88.5 ± 1.8	21	1.5	69	36.6
Clathria reinwardti (Vosmaer, 1880)	15	712 ± 146	52	85.3 ± 14.4	8	0.4	30	96.1
Clathria sp.	4	679 ± 265	143	73.8 ± 18.2	10	0.5	52	35.5
Dendroxea sp.	2	614 ± 89	265	76.7 ± 11.7	30	11.7	88	37.5
Dysidea sp.	3	1366 ± 640	409	88.1 ± 5.8	45	1.5	135	28.8
Gellioides cf. gracilis (Hentchel, 1912)	9	539 ± 93	122	82.6 ± 6.8	1	0.1	65	62.8
Gellioides sp.	4	421 ± 109	100	56.8 ± 11.5	4	2.7	83	99.4
Haliclona sp.	3	1055 ± 368	229	82.3 ± 8.2	17	1.9	120	19.6
H. (Gellius) toxotes (Hentchel, 1912)	3	718 ± 235	353	82.3 ± 3	38	3.2	91	57.6
Monanchora unguiculata (Dendy, 1922)	3	538 ± 111	228	86.6 ± 11.3	14	1.3	87	62.1
Mycale sp.	4	1236 ± 998	95	56.8 ± 6.8	8	0.2	46	79.0
Phorbas sp.	3	713 ± 251	184	87.8 ± 7.4	18	0.4	75	51.4
Pseudosuberites sp.	2	1297 ± 816	501	89.9 ± 8.4	129	3.6	125	11.6
Thrinacophora raphidophora (Hentschel, 1912)	2	1007 ± 160	473	92.6 ± 3.5	125	7.8	151	24.1

*a: Number and percentages of abundances of species-specific OTUs are calculated for the core community of each species

*b: Number and percentages of abundances of SW OTUs are calculated for the core community of each species. Values correspond to the comparison with the SW core OTUs (cosmpolitan).

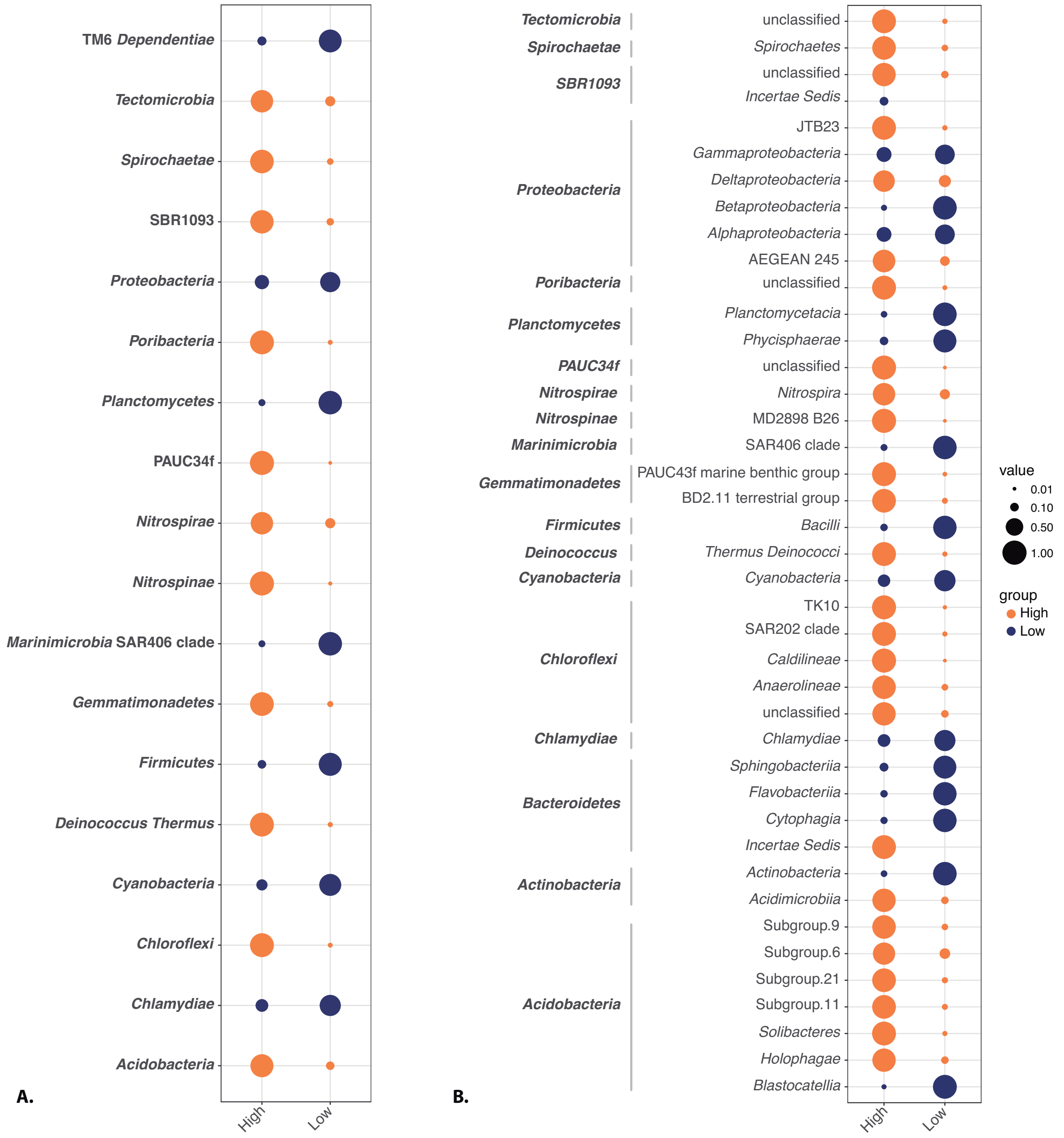

Figure S3. Indval analysis at phylum (A) and class (B) levels for bacterial taxa assignation to either HMA or LMA species groups. Only those taxa with Indval values higher than 0.6 for any of the two groups are represented. Phyla and classes significantly associated to HMA species are represented in orange and the associated to LMA species are marked in blue. Size of circles refers to the Indval value. Only the indicator classes that had abundances higher than 0.1% in each group are shown

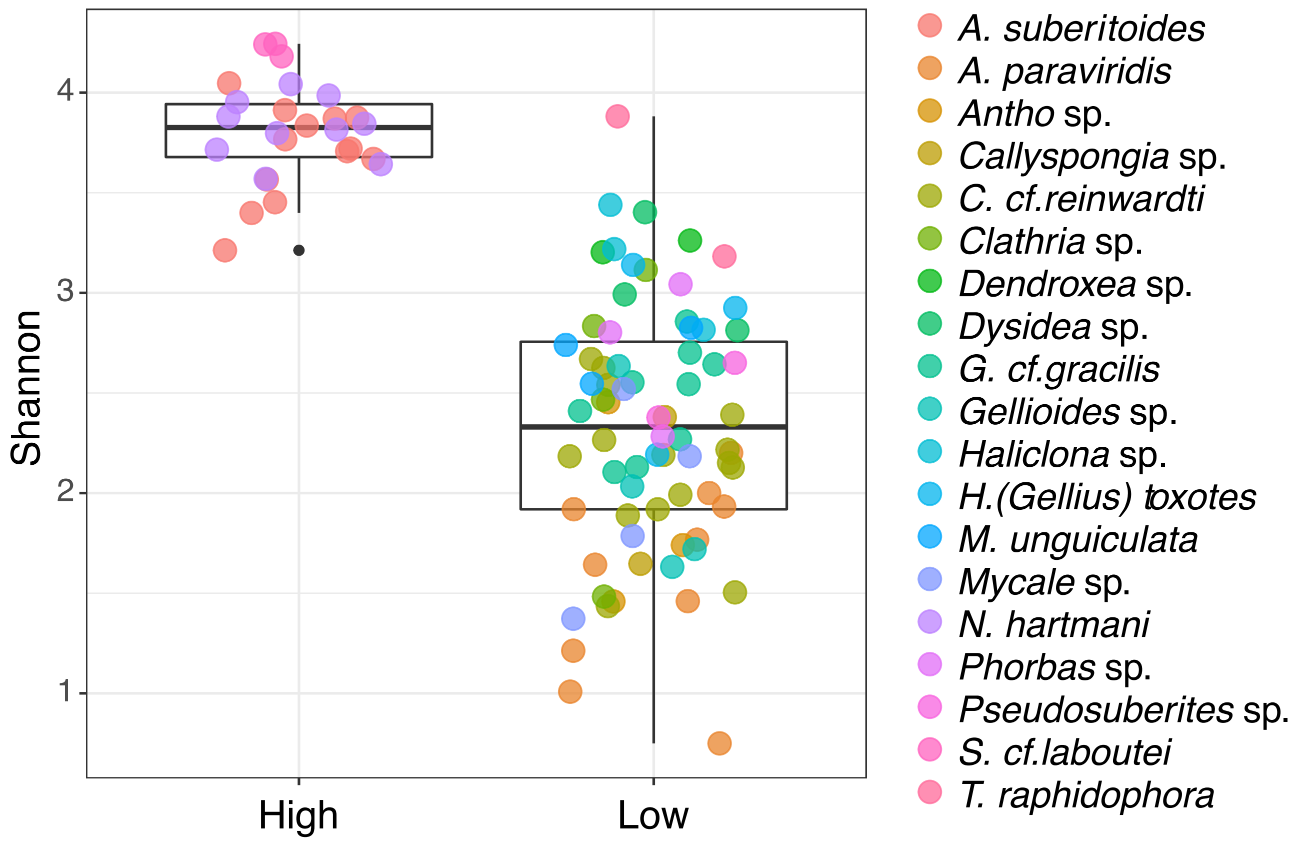

Figure S4. Boxplot showing the Shannon diversity indices of the core microbial communities for each sponge species belonging to either HMA or LMA sponge groups. Each colour corresponds to one species. Replicates of the same species are painted in the same colour.

Table S2: Percentage of sponge core shared with SW calculated for both methodologies; SW core community and abundant SW ZOTUs obtained for ZOTUs (100% seq. similarity) and OTUs (97% seq. similarity). Third column of each methodology shows the variation between both clustering methods.

Methodologies	Sponge core shared with SW core community			Sponge core shared with abundant SW ZOTUs		
Species	ZOTUs	OTUs	Variation	ZOTUs	OTUs	Variation
Aaptos suberitoides (n= 13)	48.8	56.6	7.8	2.4	3.0	0.6
Neofibularia hartmani (n= 10)	71.6	75.4	3.8	3.9	5.1	1.2
Suberea cf.laboutei (n= 3)	26.7	33.5	6.8	1.6	1.7	0.1
Amphimedon paraviridis (n= 10)	99.5	100.0	0.4	76.0	84.0	8
Antho sp. (n= 3)	9.9	11.2	1.3	4.9	8.8	3.9
Callyspongia sp. (n= 2)	38.6	36.6	-1.9	35.4	34.4	-0.9
Clathria reinwardti (n= 15)	90.4	96.1	5.7	10.9	73.6	62.7
Clathria sp. (n= 4)	33.6	35.5	1.9	23.5	24.5	1.1
Dendroxea sp. (n= 2)	34.0	37.5	3.6	27.6	31.5	3.9
Dysidea sp. (n= 3)	27.3	28.8	1.4	22.4	26.1	3.6
Gellioides cf.gracilis (n= 9)	53.0	62.8	9.7	47.4	45.9	-1.5
Gellioides sp. (n= 4)	93.2	99.4	6.2	58.5	65.8	7.3
Haliclona sp. (n= 3)	16.1	19.6	3.5	10.4	12.1	1.7
H. (Gellius) toxotes (n= 3)	60.3	57.6	-2.7	42.4	41.9	-0.5
Monanchora unguiculata (n= 3)	58.7	62.1	3.4	51.2	51.0	-0.2
Mycale sp. (n= 4)	77.5	79.0	1.5	15.8	19.5	3.7
Phorbas sp. (n= 3)	48.7	51.4	2.7	33.3	45.6	12.3
Pseudosuberites sp. (n= 2)	11.0	11.6	0.7	9.5	10.5	1.0
Thrinacophora raphidophora (n= 2)	22.7	24.1	1.5	21.9	23.5	1.6

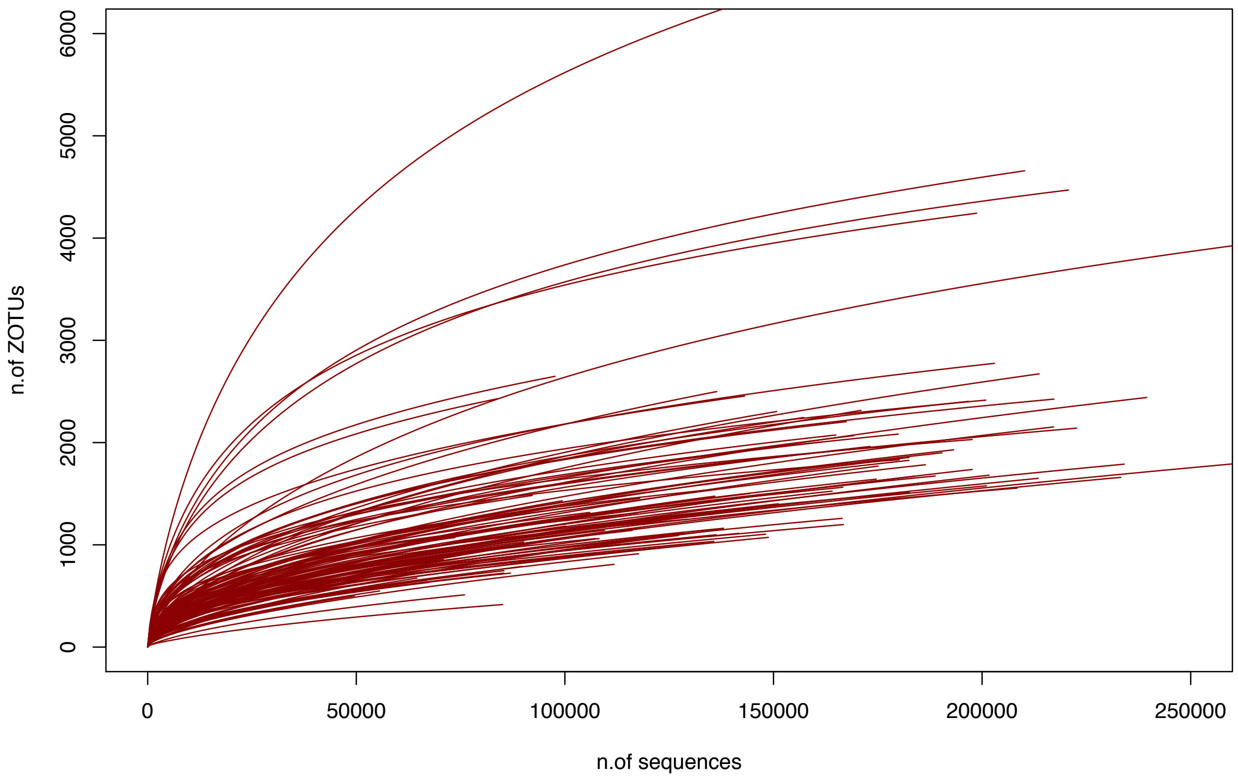

Figure S5. Rarefaction curves of all the samples used in this study. Y axis represents the number of ZOTUs and X axis represents the number of sequences. Rarefactions were performed at a minimum reads treshold of 41000.
